# The effect of antibiotic stewardship interventions with stakeholder involvement in hospital settings: a multicentre, cluster randomized controlled intervention study

**DOI:** 10.1186/s13756-018-0400-7

**Published:** 2018-09-10

**Authors:** Jannicke Slettli Wathne, Lars Kåre Selland Kleppe, Stig Harthug, Hege Salvesen Blix, Roy M. Nilsen, Esmita Charani, The Bergen Intervention Teams, Dagfinn Lunde Markussen, Andreas Thelle, Marion Iren Neteland, Ottar Hope, Ingrid Smith

**Affiliations:** 1 Department of Clinical Science, University of Bergen, Bergen, Norway; 2 Norwegian Advisory Unit for Antibiotic Use in Hospitals, Department of Research and Development, Haukeland University Hospital, Jonas Lies vei 65, N-5021 Bergen, Norway; 3 Department of Quality and Development, Hospital Pharmacies Enterprise in Western Norway, Bergen, Norway; 4 Department of Infectious Diseases and Unit for Infection Prevention and Control, Department of Research and Education, Stavanger University Hospital, Stavanger, Norway; 5 Department of Drug Statistics, Norwegian Institute of Public Health, Oslo, Norway; 6 Faculty of Health and Social Sciences, Western Norway University of Applied Sciences, Bergen, Norway; 7 NHIR Health Protection Research Unit in Healthcare Associated Infections and Antimicrobial Resistance, Imperial College, London, UK; 8 Innovation, Access and Use, Department of Essential Medicines and Health Products, World Health Organization (WHO), Avenue Appia 20, 1211 Geneva 27, Switzerland

**Keywords:** Antibiotic stewardship, Intervention, cRCT, Audit with feedback, Academic detailing, Hospital, Goal setting

## Abstract

**Background:**

There is limited evidence from multicenter, randomized controlled studies to inform planning and implementation of antibiotic stewardship interventions in hospitals.

**Methods:**

A cluster randomized, controlled, intervention study was performed in selected specialities (infectious diseases, pulmonary medicine and gastroenterology) at three emergency care hospitals in Western Norway. Interventions applied were audit with feedback and academic detailing. Implementation strategies included co-design of interventions with stakeholders in local intervention teams and prescribers setting local targets for change in antibiotic prescribing behaviour. Primary outcome measures were adherence to national guidelines, use of broad-spectrum antibiotics and change in locally defined targets of change in prescribing behaviour. Secondary outcome measures were length of stay, 30-day readmission, in-hospital- and 30-day mortality.

**Results:**

One thousand eight hundred two patients receiving antibiotic treatment were included. Adherence to guidelines had an absolute increase from 60 to 66% for all intervention wards (*p* = 0.04). Effects differed across specialties and pulmonary intervention wards achieved a 14% absolute increase in adherence (*p* = 0.003), while no change was observed for other specialties. A pulmonary ward targeting increased use of penicillin G 2 mill IU × 4 for pneumonia and COPD exacerbations had an intended increase of 30% for this prescribing behaviour (*p* < 0.001).

**Conclusions:**

Pulmonary wards had a higher increase in adherence, independent of applied intervention. The effect of antibiotic stewardship interventions is dependent on how and in which context they are implemented. Additional effects of interventions are seen when stakeholders discuss ward prescribing behaviour and agree on specific targets for changes in prescribing practice.

## Background

Globally, the overuse and misuse of antibiotics, especially broad-spectrum agents, has accelerated the development and selection of resistant bacteria [1–3]. The increase in broad-spectrum antibiotic prescribing cannot be explained by increased antibiotic resistance alone [4]. Antibiotic stewardship programs have been introduced to hospitals worldwide to promote more prudent antibiotic use [5, 6]. The basis of stewardship programs are evidence based clinical guidelines for antibiotic prescribing to ensure effective treatment for individual patients, while minimizing development of antimicrobial resistance (AMR). Adherence to antibiotic guidelines varies among countries and institutions [6]. Interventions like audit with feedback, providing a summary of clinical performance over time and educational outreach through academic detailing have been shown to be effective in increasing adherence. However, the need for studies addressing cultural, contextual and behavioural determinants when developing, implementing and reporting stewardship interventions has been highlighted [6–9]. There is also a need for more studies that apply behaviour change theory to investigate effect on antibiotic use across hospitals, specialties and diagnoses to help identify the most effective means of implementing interventions that are transferable and generalizable [6, 10, 11]. We report here the findings of a multicentre, cluster randomized controlled intervention study, investigating the effect of behaviour change interventions with stakeholder involvement and local target setting for change in antibiotic prescribing [12].

## Methods

### Definitions

Substances of ATC-group J01 (Antibacterials for systemic use), metronidazole tablets (P01AB01) and vancomycin tablets (A07AA09) were included in the definition of antibiotics for this study [13]. Broad-spectrum antibiotics were defined as penicillins with enzyme inhibitor (J01CR), 2. and 3. generation cephalosporins (J01D C-D), carbapenems (J01DH) and quinolones (J01MA), the five groups targeted in the National Action Plan Against Antibiotic Resistance in Health Services [14, 15].

### Study design

This prospective, cluster randomized, controlled intervention study was performed within three specialties at three emergency care and teaching hospitals as a parallel group study with three arms (Table 1).

**Table 1:** Overview of implemented interventions

Intervention	Hospital	Specialty	Intervention sessions	Attendance consultants/ residents (estimated)	Performed by	Special features of each intervention	Common features of intervention sessions for both audit with feedback and academic detailing
Audit with feedback	A	Pulmonary medicine	16.05.14	70%	Local pharmacist + study ward consultant	Focus areas: Pneumonia (CAP) COPD exacerbations - > Adherence to guideline (50 patients) Use of broad-spectrum antibiotics - > Indication for treatment (50 patients)	1. Verbal presentation including: - Antibiotic resistance - Antibiotic guideline - Dosing of antibiotics - Ward antibiotic sales statistics 2. Discussion among ward physicians: - Ward prescribing practice - Challenges - Target setting (1–2 targets)
	B	Infectious diseases	20.05.14 27.05.14	100%			
Academic detailing	A	Gastro-enterology	14.05.14	90%	Local ID-physician	Case discussions on recently discharged patients with infectious diseases. Variety of diagnoses, chosen by ID-physician	
	B	Pulmonary medicine	13.05.14 23.05.14	100%			
	C	Infectious diseases	20.05.14 21.05.14	70%			
Control	A	Infectious diseases					
	B	Gastro-enterology					
	C	Pulmonary medicine					

### Participants and data collection

Eligible clusters were wards within one of the medical specialties; infectious diseases, pulmonary medicine and gastroenterology at hospital A, B and C in Western Norway. Specialties were selected based on infectious diseases and pulmonary medicine having the highest consumption of antibiotics in the included hospitals. Gastroenterology was included since hospital B had a joint medication storage area for the ward of pulmonary medicine and the ward of gastroenterology. Hospital A and B were tertiary care hospitals with 1100 and 600 beds, respectively. Hospital C was a secondary care hospital with 160 beds. For description of case mix, see Table 2.

**Table 2:** Patient characteristics and patient outcome pre- and post-implementation of interventions, per intervention group and specialty

	Pulmonary medicine Period			Infectious diseases Period			Gastroenterology Period			Audit with feedback Period			Academic detailing Period			Control (all three specialities) Period		
	Pre *N* = 427 n (%)	Post *N* = 162 n (%)	*p*-value	Pre *N* = 424 n (%)	Post *N* = 153 n (%)	*p*-value	Pre *N* = 78 n (%)	Post *N* = 39 n (%)	*p*-value	Pre *N* = 478 n (%)	Post *N* = 182 n (%)	*p*-value	Pre *N* = 451 n (%)	Post *N* = 172 n (%)	*p*-value	Pre *N* = 350 n (%)	Post *N* = 169 n (%)	*p*-value
Age (mean - years)	69.6	68.99	0.66	67.5	64.4	0.10	68.9	64.8	0.35	67.1	66.1	0.51	70.2	67.1	0.05	63.2	65.7	0.22
Sex																		
Female	213 (49.9)	88 (54.3)	0.34	180 (42.5)	80 (52.3)	**0.04**	37 (47.4)	16 (41.0)	0.51	221 (46.2)	101 (55.5)	**0.03**	209 (46.3)	83 (48.3)	0.67	167 (47.7)	80 (47.3)	0.94
Male	214 (50.1)	74 (45.7)		244 (57.5)	73 (47.7)		41 (52.6)	23 (59)		257 (53.8)	81 (44.5)		242 (53.7)	89 (51.7)		183 (52.3)	89 (52.7)	
Admitted from hospital/nursing home	55 (12.9)	15 (9.3)	0.23	68 (16.0)	30 (19.7)	0.30	14 (18.0)	3 (7.7)	0.14	67 (14.0)	20 (11.0)	0.30	70 (15.5)	28 (16.4)	0.79	51 (14.6)	23 (13.6)	0.75
Indication for antibiotic treatment																		
Pneumonia	136 (31.9)	63 (38.9)	0.20	115 (27.1)	45 (29.6)	0.06	11 (14.1)	3 (7.7)	0.16	162 (33.9)	67 (36.8)	**0.01**	100 (22.2)	44 (25.7)	0.15	65 (18.7)	23 (13.6)	0.31
COPD exacerbation, infectious	183 (42.9)	63 (38.9)		59 (13.9)	7 (4.6)		1 (1.3)	2 (5.1)		132 (27.6)	34 (18.7)		111 (24.6)	38 (22.2)		25 (7.2)	15 (8.9)	
LRTI - Other	23 (5.4)	9 (5.6)		16 (3.7)	6 (3.95)		6 (7.7)	2 (5.1)		19 (4.0)	4 (2.2)		26 (5.8)	13 (7.6)		22 (6.3)	7 (4.1)	
Sepsis	40 (9.4)	17 (10.5)		61 (14.4)	24 (15.8)		16 (20.5)	5 (12.8)		57 (11.9)	26 (14.3)		60 (13.3)	20 (11.7)		73 (21.0)	50 (29.6)	
Skin and soft tissue	3 (0.7)	2 (1.2)		62 (14.6)	33 (21.7)		9 (11.5)	6 (15.4)		31 (6.5)	25 (13.7)		43 (9.5)	16 (9.4)		58 (16.7)	22 (13.0)	
Gastrointestinal tract	1 (0.2)	0 (0)		9 (2.12)	4 (2.6)		9 (11.5)	11 (28.2)		6 (1.3)	2 (1.1)		13 (2.8)	13 (7.6)		35 (10.1)	14 (8.3)	
Urinary tract	9 (2.1)	5 (3.1)		75 (17.7)	21 (13.8)		17 (21.8)	9 (23.1)		31 (6.5)	16 (8.8)		70 (15.5)	19 (11.1)		36 (10.3)	19 (11.2)	
Other	32 (7.5)	3 (1.9)		27 (6.4)	12 (7.9)		9 (11.5)	1 (2.6)		40 (8.4)	8 (4.4)		28 (6.2)	8 (4.7)		34 (9.8)	19 (11.2)	
Antibiotic allergies	62 (14.5)	28 (17.3)	0.41	35 (8.3)	19 (12.4)	0.13	8 (10.3)	4 (10.3)	0.99	59 (12.3)	26 (14.3)	0.51	46 (10.2)	25 (14.5)	0.13	25 (7.2)	17 (10.1)	0.18
Patient outcomes ∆																		
Length of stay (days)	7.2	6.9	0.40	6.5	6.5	0.99	7.0	6.1	0.30	7.2	6.5	**0.04** ^**∆**^	6.5	6.8	0.41	7.5	6.8	0.11
In-hospital mortality	30 (7.0)	7 (4.3)	0.23	12 (2.8)	4 (2.61)	0.89	3 (3.9)	2 (5.13)	0.76	28 (5.9)	9 (5.0)	0.65	17 (3.8)	4 (2.3)	0.37	13 (3.7)	3 (1.8)	0.23
30-day mortality	53 (12.4)	11 (6.8)	0.05	30 (7.1)	8 (5.3)	0.44	3 (3.9)	4 (10.3)	0.17	50 (10.5)	12 (6.6)	0.13	36 (8.0)	11 (6.4)	0.51	24 (6.9)	6 (3.6)	0.13
30-day readmission	117 (29.3)	38 (24.5)	0.26	78 (19.1)	18 (12.2)	0.06	16 (21.3)	6 (17.1)	0.61	111 (24.6)	36 (20.9)	0.33	100 (23.1)	26 (15.7)	**0.04** ^**∆**^	58 (17.5)	30 (18.6)	0.75

*p*-values are obtained by chi-square test (categorical variables) and t-test (continuous variables) per group

∆ Patient outcomes were also tested for interaction between intervention groups/specialty and period using linear and logistic regression. *p*-value interaction length of stay = 0.080. *p*-value interaction 30-day readmission = 0.105

Patients who received antibiotics during hospitalization and were discharged from the study wards in the time period from 10th of February to 11th of July 2014 were eligible for inclusion in the study. Patients who received antibioticprophylaxis, had orthopaedic prosthesis infections, or had a hospital stay < 24 h or > 21 days were excluded. Patients whose indication for treatment was not in the antibiotic guideline or whose antibiotics were discontinued at day 1, was excluded. Only the first stay of readmitted patients was included. Patients were included consecutively. Patient data were collected manually from electronic medical records. Data collected included patient demographics, indication for antibiotic treatment, antibiotic prescribing, microbiological test results, estimated glomerular filtration rate (eGFR) on admission, length of stay, 30-day readmission, in-hospital and 30-day mortality and admittance from- or discharge to other hospitals or nursing homes. Indications for antibiotic treatment were registered as documented in the medical record and not assessed for validity.

Broad-spectrum antibiotic use for study wards in the period 2013–2015 was collected from the hospital pharmacies sales statistics and adjusted per 100 patient bed days.

### Interventions

The primary intervention aim was to increase adherence to The National Guidelines for Antibiotic Use in Hospitals (hereafter guidelines), across diagnoses [16]. Each hospital assigned local intervention teams of 1–2 physicians and 1 pharmacist to co-design and implement the interventions. Authors I.S and J.S.W developed initial intervention concepts, which were discussed in a regional meeting with all project participants. Each intervention team then refined the interventions to fit their local context. A common presentation template was prepared for all intervention sessions with information about antibiotic resistance, the national antibiotic guideline, local antibiotic sales statistics and principals of antibiotic dosing. All intervention teams modified this material to fit the individual wards. Academic detailing sessions focused on recently admitted infectious diseases patients, including cases with treatment both adherent and non-adherent to guidelines. The teams’ selection of patient cases decided the focus in wards receiving academic detailing.

Audit with feedback wards had predefined target areas of pneumonia and COPD exacerbations, as these patients were frequently admitted to both intervention wards. Fifty patients with these diagnosis were included consecutively from February to April 2014 to get a reasonable overview of prescribing practice over the given time period, without excessive workload for the intervention teams. For the audit data, intervention teams assessed adherence. The level of detail and focus in the feedback was at the discretion of the teams and varied between the two feedback wards.

Intervention ward physicians were invited to academic detailing- or audit with feedback- group sessions in May 2014, led by local intervention teams. No specific threshold for acceptable attendance was defined, but more than one meeting was held if the intervention team considered the attendance at the first meeting to be too low. Physicians present at the main session at each ward were invited to identify one or two specific challenges to be addressed as local targets for improvement of antibiotic prescribing based on discussions during the session. Specific actions to achieve targets were not included in the target discussions. For details of interventions, see Table 1.

### Outcomes

#### Primary outcome measures

1. Adherence to guidelines was assessed on the second day of treatment to allow sufficient time for patients to be reviewed by study ward physicians and measured as percentage of correctly prescribed empiric treatment (choice of active substance) before and after interventions [16]. CRB-65 was not routinely documented, so pneumonia and severe pneumonia was assessed together (both empiric treatments assessed as adherent). All hospitals were committed to use the national guideline, as recommendations were appropriate with regards to local antibiotic resistance patterns.
2. Use of broad-spectrum antibiotics was assessed as DDD/100 bed days in time series before and after intervention. Broad-spectrum antibiotic use was selected as an outcome measure because the guidelines mainly recommend narrow-spectrum antibiotics as empiric treatment and a shift towards guideline adherent prescribing was expected to cause a reduction in broad-spectrum antibiotic use.
3. Change in locally targeted prescribing behaviour was assessed according to the defined targets and compared before and after interventions.

*Secondary outcome measures* were length of stay, 30-day readmission and mortality (all cause in-house and 30-day mortality). Patient outcomes were measured to ensure that the interventions did not have any negative consequences for patient treatment.

#### Sample size

As baseline adherence to guidelines was unknown in Norway, calculation of the sample size prior to the study was challenging. According to the original research protocol, we assumed an absolute 20% improvement in adherence from 50% pre-intervention to 70% post-intervention for each cluster. Given a power of 80% and a type 1 error of 5%, the smallest number of subjects needed to detect this difference was 93 both before and after the intervention. Although this was sufficient for the current study, we calculated at least 155 patients before and after intervention to answer additional research questions listed in the original protocol. However, the sample size calculations did not include intra-cluster correlation coefficient (ICC) or comparison with a control group. Based on pre-intervention data of adherence to guideline, ICC coefficient for this outcome was 0.012 with 95% CI (0.003, 0.053).

#### Randomization

Authors I.S and J.S.W. performed randomization and assigned clusters to interventions by drawing lots of hospital and intervention groups per specialty. Across the hospitals, infectious diseases and pulmonary medicine received both academic detailing and audit with feedback and had a control group. Only two of the hospitals had specific gastroenterology wards, so this specialty received only one intervention and had a control group (Table 1).

#### Blinding

Prescribing physicians at the wards were not informed about the study being performed during the baseline period and were at that point blinded to intervention group, with the exception of the physicians assigned to the project teams. Control ward physicians were blinded throughout the study period.

Assessment of adherence to guidelines was performed blinded to the intervention- or treatment group, by using syntax in SPSS. An adherence variable was generated, combining the variable indication for treatment with the variable for prescribed treatment. First choice of empiric therapy was coded as adherent. Manual adjustment of adherence of antibiotic prescriptions was made in patients with antibiotic allergies or kidney failure.

#### Statistical analysis

Analyses were performed both per intervention group and per specialty, but due to fewer patients than expected in the post-intervention period, analysis per cluster was not performed. Differences in study group characteristics pre- and post-interventions were tested using Pearson’s chi-square test for categorical data and independent two-sample t-test for continuous data. Pearsons chi-square test was also applied to test adherence to guidelines pre- and post-interventions for individual intervention groups and specialties. To test whether percentage of adherence to guidelines or patient outcomes in intervention and specialty groups changed differently over time compared with the control group, we evaluated the group-by-period interaction term in simple logistic or linear regression models, as appropriate. Adherence to guideline or patient outcome were dependent variables, with group of intervention (audit vs control/academic detailing vs control) or specialty (e.g. pulmonary medicine vs control/infectious diseases vs control) and period (before-after) were independent variables together with the interaction term. The level – and trend effect of broad-spectrum antibiotic use (sales statistics) pre- and post-intervention was estimated with the Interrupted Times Series (ITS) analysis method described by the Cochrane Effective Practice and Organisation of Care (EPOC) group [17]. All tests were two-sided and *p*-values < 0.05 was considered statistical significant for all analyses. Statistical analysis was conducted using SPSS for Windows, version 24 and Stata SE for Windows, version 15.

## Results

### Patients

Two thousand four hundred five admissions were eligible for inclusion. After applying exclusion criteria, 1802 unique patients were included in analysis, 1279 and 523 patients in the pre- and post-intervention periods respectively (Table 2). The study period was fixed due to time-limited allocation of project resources and mandatory information of included patients. Interventions were conducted later than originally planned due to practical considerations at the study wards. This caused skewness in data with two thirds of the patients included pre-interventions (Table 2). Patient characteristics were similar pre- and post-interventions, except for some differences in distribution of diagnoses in the audit with feedback group (Table 2).

### Primary outcomes

#### Adherence to guidelines

Across all intervention wards, adherence to guideline increased from 60% to 66% (*p* = 0.04), but when compared with the control group, this was not significant (Table 3). The effect of interventions differed largely between the specialties. Infectious diseases and gastroenterology wards displayed no effect of interventions on adherence, while pulmonary medicine wards displayed significant effect of interventions compared to the control group (Table 3). Academic detailing and audit with feedback increased total adherence to guideline by 14% and 13% respectively (absolute increase), in the pulmonary wards (not shown in tables).

**Table 3:** Percentage of adherence to antibiotic guidelines in periods before and after interventions were implemented

Group	Group description	N Before/after	Period		Absolute Change %	P for change^a^	P for Interaction^b^
			Before n (%)	After n (%)			
Intervention							
Control	All specialties	350/169	174 (50)	84 (50)	0	0.998	
Interventions	All specialties	929/354	556 (60)	234 (66)	6	**0.04**	0.252
Academic detailing	All specialties	451/172	265 (59)	111 (65)	6	0.188	0.353
Audit with feedback	Infectious diseases + Pulmonary medicine	478/182	291 (61)	123 (68)	7	0.111	0.265
Specialty							
Pulmonary medicine	Both interventions	427/162	249 (58)	116 (72)	14	**0.003**	**0.034**
Infectious diseases	Both interventions	424/153	268 (63)	99 (65)	2	0.741	0.857
Gastroenterology	Academic detailing	78/39	39 (50)	19 (49)	-1	0.896	0.556

^a^By chi-square test per group

^b^By logistic regression of given group vs control wards (all specialties), giving the *p*-value for the interaction between group and period

*P*-values < 0.05 are given in boldface

The audit with feedback intervention specifically targeted pneumonia and COPD exacerbations. For these diagnoses, the pulmonary medicine ward increased adherence by 12% and infectious diseases ward by 2% (not shown in tables).

#### Use of broad-spectrum antibiotics

**Fig. 1:**
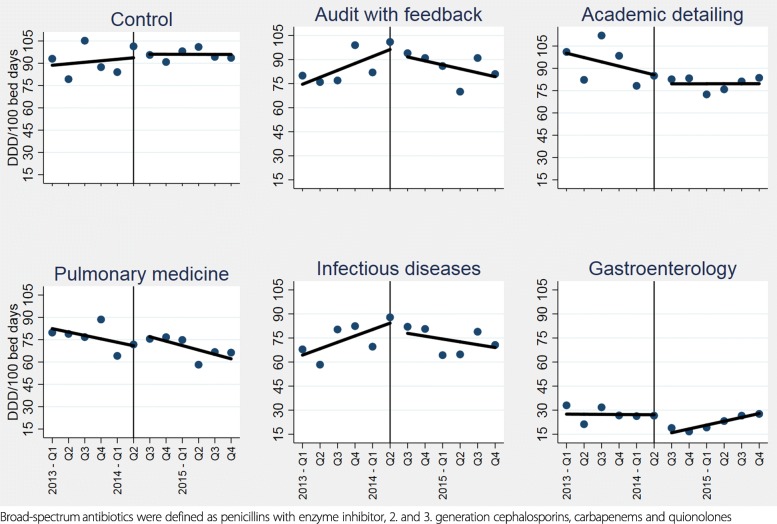
Use of broad-spectrum antibiotics at intervention wards and per specialty intervention wards compared to control wards

Interrupted time series analysis showed that the overall trend of activity-adjusted broad-spectrum antibiotic use pre- and post-interventions was significantly improved, as was the level at 12 and 18 months post intervention for the audit with feedback group (Appendix: Table 5 and Fig. 1). The gastroenterology intervention ward had a significant decrease in the use of broad-spectrum antibiotics at 3 and 6 months, but it increased thereafter (Appendix: Table 5 and Fig. 1). No significant change in broad-spectrum antibiotic use was seen at the intervention wards receiving academic detailing, the control group and for other intervention wards per specialty (Appendix: Table 5 and Fig. 1).

#### Local targets

Intervention wards were invited to set local targets for follow up after the intervention sessions (Table 4). The pulmonary ward at Hospital A had a significant and intended 30% increase in the targeted use of Penicillin G 2 mill IU × 4 for patients with pneumonia and COPD exacerbations post intervention (*p* < 0.001). The use of Ciprofloxacin at the ward of gastroenterology was reduced at all time points following the intervention, though not statistically significant (Appendix: Table 6). The other study wards either ^a)^ did not reach consensus on targets ^b)^ did not identify any targets or ^c)^ the identified target was not evaluable.

**Table 4:** Local targets set by study intervention wards and outcome for targeted change in prescribing practice

Hospital	Ward	Intervention	Targets	Outcome
A	Pulmonary medicine	Audit with feedback	Increase the use of Penicillin G 2 mill IU × 4 to treat pneumonia (CAP) and infectious COPD exacerbations	30% increase (*p* < 0.001)^a^
A	Gastroenterology	Academic detailing	Reduce ciprofloxacin use for inflammatory bowel disease, and shift to Co-trimoxazol *(indication outside national antibiotic guideline)*	Too few patients with targeted indication to assess outcome by indication. Assessed by use of sales statistics. Reduction in use of Ciprofloxacin at 3, 6, 12 and 18 months following the intervention (not significant)^b^ (Appendix Table 6).
B	Pulmonary medicine	Academic detailing	Target areas discussed: - Reevaluation of initiated treatment on arrival to ward and - after 48–72 h - Increase use of CRB-65 and antibiotic guideline	*Consensus on 1–2 targets not achieved*
B	Infectious diseases	Audit with feedback	Target areas discussed: - Increase use of Penicillin G 2 mill × 4 to treat infectious COPD exacerbations - Reassess length of iv-antibiotics for patients with osteomyelitis - Increase consultants presence in the emergency room to increase guidelines adherence on admission - Reevaluation of treatment during the patient stay	*Consensus on 1–2 targets not achieved*
C	Infectious diseases	Academic detailing	*No target area identified.*	*No target area identified*

^a^By chi-square test ^b^By Interrupted time series analysis (Appendix Table 6)

### Secondary outcome measures

When analysed per intervention, there was a decrease of 0.7 days in the mean length of stay for patients in the audit with feedback group (*p* = 0.037) (Table 2). In the academic detailing group, 30-days readmission had an absolute decrease of 7.4% (*p* = 0.044). Compared with the control group, these findings were not statistically significant. In-hospital death, 30-day mortality, 30-day readmission and length of stay for the other groups were not significantly changed (Table 2).

## Discussion

This study highlights the effect of engaging local stakeholders (physicians) in setting specific targets for change in antibiotic prescribing behaviours. A specific target area, which is easy to remember and act upon, makes it possible to achieve change within a short timeframe, as observed in the pulmonary ward at Hospital A where adherence to targeted behaviour increased by 30%. Another finding was how the effect of interventions differed across specialties. Both interventions were more effective at the pulmonary wards, than wards of infectious diseases and gastroenterology.

Interventions to improve antibiotic prescribing practices have shown a 15% average increase in adherent prescribing in intervention wards, however the effect depends on how they are designed and implemented [6, 18]. When Schouten et al. tailored interventions to each intervention hospital; they achieved an average 14% increase in adherence to guidelines for empiric treatment of lower respiratory tract infections (LRTI). The level of change was very similar for all the intervention hospitals, although it was not stated how the patients were distributed across the wards of internal- and pulmonary medicine [19]. Their results are comparable to our findings at the pulmonary wards, with a 14% absolute change in adherence to guidelines, while it differs substantially from effects seen across infectious diseases and gastroenterology wards. A single site Norwegian study focusing on pneumonia and COPD exacerbations within pulmonary medicine, added a pocket guideline to their audit with feedback intervention [20]. From a baseline adherence of 62%, similar to our study, adherence was increased by 22%.

**Involving clinicians** in identifying challenges, finding solutions and setting local targets is both reasonable and recommended and has previously proven effective in increasing compliance to target behaviour [6, 21–23]. Jobson et al. increased the timeliness of antibiotics for febrile patients with central lines presenting in the ED from 63 to 99% [23]. They exceeded their goal of 90% timeliness through active engagement of the caregiving staff and the use of multiple plan-study-do-act-cycles (PDSA-cycles) [24]. At the pulmonary ward at hospital A, the audit data made it easy for clinicians to identify local challenges and set a specific, measureable, attractive and realistic target for change in prescribing behaviour and we found a similar change of 30% increase in target behaviour.

The wards receiving audit with feedback had different case-mix. In infectious diseases, 41% of patients were treated for pneumonia and COPD exacerbations, compared to 71% in the pulmonary ward. This could partly explain the lack of effect seen in the infectious disease ward, as pneumonia and COPD exacerbations were the selected focus for the feedback sessions. Empirical therapy according to guidelines across diagnoses was the main outcome measure for all intervention wards. Pre-audits at every intervention ward would have made it easier to identify each ward’s prescribing challenges, and tailor the interventions to context specific improvement areas for each ward. As this study is intervening in the very heart of ID-specialists’ area of expertise, it may also be a bigger challenge to advocate a shift in prescribing practice towards general antibiotic guidelines, limiting the autonomy of the prescriber [25].

The national guideline was published approximately 6 months prior to study initiation and some wards had already started promoting its use [16]. A previous study by Skodvin et al. showed that interns and residents heavily relied on guidelines when initiating antibiotic treatment [26]. This could have caused a positive shift in prescribing practice already, decreasing the potential for absolute effect of interventions. Including physicians mainly working in the emergency room in interventions could have given increased effects, but intervention and control wards at the same hospital would then be challenging because of spill over effects between the wards.

Champions can play a powerful role in behaviour change [25, 26]. Special emphasis was made on using local champions for developing and implementing interventions as they are familiar to the ward physicians, know possible barriers and facilitators and could tailor the presentations to the ward’s needs. Local involvement could also increase the chance of continuous work within the area after study completion. An example of tailoring is adding information about a previous local outbreak of Vancomycin-resistant enterococci (VRE) to the audit with feedback session at the pulmonary ward at Hospital A, to increase local ownership. At the gastroenterology ward, academic detailing was performed by an ID-physician. During evaluation, he suggested that including a physician from the gastroenterology ward could have increased the ownership of the intervention and the identified target. Interventions were only applied at one time-point during the study period. Adding more intervention sessions could probably have increased the effects seen [6].

We aimed to achieve responsible antibiotic prescribing practice in a complex hospital setting. This study is a “real-life” study, including the most common infections treated at hospitals in the western world and three specialties in three separate hospitals where patterns of prescribing may differ, as will patient mix. All three hospitals and specialties contributed both to the intervention and control groups, reducing the potential for confounding and increasing external validity of the findings. The study was initiated in a “normal” clinical situation and not as a response to an outbreak. Random time effects should therefore be reduced. Seasonality is likely, but the inclusion of control groups within the same time period allow us to control for the effects. Findings should be generalizable to other hospital wards within the same specialties and in settings with a similar, relatively flat organizational structure.

The short post-intervention period and skewness of data between pre- and post-intervention periods is the major limitation to this study, caused by the fixed date for study period when applying for study approval and the substantial workload for manual data collection of individual prescription data. This also led to insufficient power to look at intervention effect on adherence at each cluster. Activity-adjusted antibiotic sales statistics for broad-spectrum antibiotics provides however the opportunity to assess change in levels and trends of broad-spectrum antibiotic use, indicating prescribing behaviour over longer periods of time.

In our study we found that the context we implemented interventions in were even more important than the type of intervention selected. Tailoring the interventions to the local context and challenges of each study ward and more focus on using SMART^1^ goals during the planning and implementation of interventions, could increase the possibility to get the desired outcomes. LRTIs are common in stewardship intervention studies [6, 18]. It is a wise place to start optimization of antibiotic prescribing, because the volume of patients secures great impact on total antibiotic use. More severe diagnoses, like infections in immunocompromised patients may be a bigger challenge to target in behaviour change. Especially inexperienced physicians may feel the need to secure adequate coverage with broad-spectrum antibiotics at treatment initiation and the thought of “never change a winning team” may lead to lack of re-evaluation and focusing treatment [26].

When designing behavioural change interventions in antibiotic stewardship programs, we need careful planning. Attention should be paid to local barriers and facilitators for change and we should have in-depth knowledge of local antibiotic prescribing practices and case mix to guide the focus of interventions.

## Conclusions

Pulmonary intervention wards had an increase in adherence, independent of applied intervention, while no effect was seen at wards of infectious diseases and gastroenterology. This shows that the context in which interventions are implemented is important and may also indicate that pulmonary wards may be a good place to start when changing antibiotic prescribing behavior in similar hospital settings. We also showed that when ward physicians were actively involved in the process of discussing their own prescribing behavior and could identify and agree on specific targets for change in prescribing practice, great change was achieved within a short timeframe.

## Appendix

Appendix show results for interrupted time series analysis of the use of broad- spectrum antibiotics for intervention groups and specialties:

**Table 5:** Interrupted time series analysis of the use of broad-spectrum antibiotics in defined daily doses per 100 bed days for intervention and control groups from 2013 to 2015

		Audit with feedback (Intervention wards)			Academic detailing (Intervention wards			Pulmonary medicine (Intervention wards)			Infectious diseases (Intervention wards)			Gastroenterology (Intervention ward)			Control (all specialties)		
		Estimate	SE	*p*-value	Estimate	SE	*p*-value	Estimate	SE	*p*-value	Estimate	SE	*p*-value	Estimate	SE	*p*-value	Estimate	SE	*p*-value
DDD per 100 bed days	Const	70.288	5.854	0.000	103.078	8.415	0.000	84.736	5.056	0.000	60.371	7.407	0.000	27.605	1.592	0.000	87.691	5.387	0.000
	AR	−0.495	0.326	0.172	−0.247	0.370	0.525	−0.429	0.348	0.257	−0.239	0.372	0.542	−0.796	0.203	0.006	−0.512	0.323	0.157
Pre-slope		4.332	1.523	**0.025**	−2.903	2.181	0.225	−2.292	1.312	0.124	3.985	1.926	0.077	−0.075	0.411	0.861	1.002	1.404	0.498
Difference between pre- and post-slope		−6.789	2.028	**0.012**	2.920	2.985	0.360	−0.669	1.770	0.717	−5.753	2.615	0.064	2.472	0.545	**0.003**	−1.033	1.863	0.597
Level effect																			
3 months		−8.971	7.809	0.288	−3.162	11.053	0.783	8.314	6.686	0.254	−10.360	9.839	0.327	−11.157	2.082	**0.001**	1.407	7.191	0.850
6 months		−15.761	8.313	0.100	−0.242	11.823	0.984	7.646	7.131	0.319	−16.112	10.503	0.169	−8.685	2.220	**0.006**	0.374	7.672	0.962
12 months		−29.338	10.490	**0.027**	5.599	15.115	0.722	6.308	9.052	0.508	−27.617	13.357	0.077	−3.740	2.811	0.225	−1.691	9.694	0.866
18 months		−42.916	13.561	**0.016**	11.439	19.708	0.580	4.971	11.751	0.685	−39.122	17.355	0.059	1.205	3.640	0.750	−3.757	12.525	0.773

Broad-spectrum antibiotics were defined as penicillins with enzyme inhibitor, 2. and 3. generation cephalosporins, carbapenems and quionolones

**Table 6:** Interrupted Time Series analysis of the use of Ciprofloxacin at the ward of Gastroenterology, Hospital A, from 2013 to 2015

		Estimate	SE	*p*-value
Ciprofloxacin DDD per 100 bed days	Constant	10.291	1.283	0.000
	AR	−0.090	0.520	0.868
Pre-slope		−0.169	0.341	0.636
Difference between pre- and post-slope		−0.106	0.461	0.825
Level effect				
3 months		−4.254	1.905	0.061
6 months		−4.360	1.956	0.061
12 months		−4.571	2.343	0.092
18 months		−4.783	2.975	0.152

Use of Ciprofloxacin is measured as quarterly sales of Ciprofloxacin, adjusted for bed days

## Abbreviations

COPD: Chronic obstructive pulmonary disease
CRB-65: Score of community acquired pneumonia assessing: Confusion – Respiratory rate – Blood pressure – Age (65-years of age or older)
eGFR: estimated Glomerular Filtration Rate
EPOC: Cochrane Effective Practice and Organization of Care
ITS: Interrupted time series
LRTI: Lower respiratory tract infection
SMART goals: Specific – Measurable – Achievable/Attractive – Realistic – Time-bound

## Footnotes

1 SMART: Specific, Measurable, Attractive, Realistic and Time-bound
